# The Effects of Feed Restriction and Isolated or Group Rearing on the Measurement of Individual Feed Intake and Estimation of Feed Conversion Ratio in Juvenile Nile Tilapia (*Oreochromis niloticus*) for Selective Breeding Purposes

**DOI:** 10.3389/fgene.2020.596521

**Published:** 2021-01-15

**Authors:** Charles Rodde, Marc Vandeputte, Trong Quoc Trinh, Vincent Douchet, Marc Canonne, John A. H. Benzie, Hugues de Verdal

**Affiliations:** ^1^CIRAD, UMR ISEM, Montpellier, France; ^2^ISEM, Université Montpellier, CNRS, EPHE, IRD, Montpellier, France; ^3^WorldFish, Bayan Lepas, Malaysia; ^4^MARBEC, CNRS, Ifremer, IRD, University of Montpellier, Montpellier, France; ^5^Université Paris-Saclay, INRAE, AgroParisTech, GABI, Jouy-en-Josas, France; ^6^School of Biological, Earth and Environmental Sciences, University College Cork, Cork, Ireland

**Keywords:** feed efficiency, individual rearing, group rearing, fish, video analyses, feed intake

## Abstract

Accurately measuring the phenotype at the individual level is critical to the success of selective breeding programs. Feed efficiency is a key sustainability trait and is typically approached through feed conversion ratio (FCR). This requires measurements of body weight gain (BWG) and feed intake (FI), the latter being technically challenging in fish. We assessed two of the principal methods for measuring feed intake in fish over consecutive days: (1) group rearing 10 fish per group and video recording the meals and (2) rearing fish individually on a restricted ration. Juvenile Nile tilapia (*Oreochromis niloticus*) from the Genetically Improved Farmed Tilapia (GIFT) strain and the Cirad strain were entered into the study (128 GIFT and 109 Cirad). The GIFT strain were reared over three consecutive periods of 7 days each under different feeding, recording, and rearing scenarios (i) in groups fed an optimal ration (g100) or (ii) fed a 50% restricted ration (g50) both with video records of all meals and (iii) reared in isolation and fed a 50% restrictive ration. The Cirad strain were tested similarly but only for scenarios (i) and (iii). All fish were fed twice daily with a calculated ration. Correlations showed the same trends for the GIFT and the Cirad strains. For the GIFT strain, correlations were positive and significant for BWG and FI measured in scenarios (i) and (ii), 0.49 and 0.63, respectively, and FI measured in scenarios (i) and (iii) (0.50) but not for BWG measured in scenarios (i) and (iii) (0.29, NS). The phenotypic correlation estimated for FCR between scenarios (i) and (ii) with fish fed an optimal or a 50% restricted ration was low and not significant (0.22). Feed Conversion Ratio for GIFT fish reared in groups or in isolation and fed with a restricted ration [scenarios (ii) and (iii)] were not significantly correlated either. Social interactions between fish, potentially impacting their efficiency, may explain the results. Therefore, selective breeding programs seeking to improve feed efficiency will need to carefully plan the feeding rate and the rearing system used to estimate FCR in order to optimize selection for the targeted production system.

## Introduction

In aquaculture, feed represents 30–70% of farm costs (Doupé and Lymbery, 2004; de Verdal et al., 2018a) and is the primary expenditure of intensive fish farming (Rana et al., 2009). Several ways have been investigated to improve the use of feed by fish, including nutrition (Reigh and Ellis, 1992; Gaylord and Gatlin, 2001; Yao et al., 2014), husbandry (Alanärä, 1996; Imsland et al., 2005; Yilmaz and Arabaci, 2010), and genetics (Kause et al., 2006b; de Verdal et al., 2018b; Knap and Kause, 2018; Besson et al., 2020). While nutrition and husbandry have been widely studied and applied in production, genetic approaches need more investigation to enable practical implementation. A selective breeding program to improve feed efficiency typically involves recording of feed intake (FI) which has to be accurately measured at the individual level in order to calculate feed conversion ratio (FCR) which is the ratio between the feed consumed by a fish and its growth during the same period of time [FCR = feed intake/body weight gain (BWG)]. This is particularly challenging for fish as they are reared in water and generally in large groups. The most commonly used method of the few developed to date has been the X-ray method. This uses radio-opaque glass beads included in the feed pellets allowing an assessment of how much feed the fish have ingested (Talbot and Higgins, 1983; McCarthy et al., 1993; Jobling et al., 2001; Silverstein et al., 2001; Kause et al., 2006a; Grima et al., 2008). However, while this method is accurate to monitor feed intake in a one specific meal, the repeatability of FI measurement is relatively low (Kause et al., 2006a; Grima et al., 2008) and it is not possible to measure FI of several consecutive meals due to the recovery time needed between two measurements. In genetic studies, even with repeated measurements (five measurements at 2 weeks intervals), heritability of FCR remains low (<0.07) in whitefish, suggesting the existence of significant residual environmental variance (Quinton et al., 2007).

As the FI of an individual fish in consecutive days is highly variable (Jobling and Koskela, 1996; de Verdal et al., 2017), the ideal method to measure individual feed efficiency should allow the measurement of FI over several consecutive days, so that amount of feed eaten by a fish over a period where it achieves significant growth is known with a high accuracy.

Recently, two alternative methods have been developed which can be upscaled to meet the needs of recording hundreds of individuals for genetic studies and which overcome the constraints of between day variability in feed intake. The first one is individual rearing of fish in aquaria fed a known restricted feed ration, combined with precise daily counting of uneaten pellets (Besson et al., 2019). Using this method, fish can be reared for a few weeks or months, and FI can be measured accurately over a long period of time. An important aspect of this method is that fish are fed under a restricted ration, leading to a strong correlation of FCR with growth as individuals cannot express their own variability for satiety level (Henryon et al., 2002). This can be an advantage, as simple selection for growth under restricted feeding can lead to improvement in feed efficiency, which are suggested in fish (Besson et al., 2019) and well-proven in rabbits and pigs (Nguyen et al., 2005; Drouilhet et al., 2016). Another advantage of restricted feeding is that the amount of uneaten pellets to be removed and counted every day is reduced compared to what would happen under satiation feeding, making the workload more compatible with the evaluation of hundreds of fish (Besson et al., 2019). However, restricted feeding may be problematic because the FCR expressed in this condition may differ from that under satiation feeding. Also, as fish are reared in isolation, they lose all the social interactions between each other, and this can have a high impact on performance.

The second method, developed some time ago (see review by Jobling et al., 2001) and adapted to genetic studies by de Verdal et al. (2017) consists of rearing small groups of fish in aquaria (ten to 15 fish together) and to video-record all the meals, pellets being provided one by one in several different places in the aquarium to reduce competition between fish. Using this methodology and having a visible identification of all the fish in the aquarium, it is possible to count the number of pellets eaten by each individual fish, and consequently, to estimate their feed intake. Measurement of FI using this method is accurate, the feed ration can be optimal (no need for any restriction), it permits social interactions between the fish during all the rearing period, but it is time-consuming, as it is necessary to analyse all the videos of all the meals.

When used with family designs in fish, both methods produced comparable heritability estimates: 0.47 for FCR in European sea bass (*Dicentrarchus labrax*) with the isolation method using restricted ration and genomic information (Besson et al., 2019), and 0.32 for FCR in Nile tilapia (*Oreochromis niloticus*) with the video method using pedigree information (de Verdal et al., 2018b). However, these two methods (isolation with a restricted feed ration *vs*. in groups with an optimal feed ration) have very different approaches. There is presently no evidence of correlations between feed efficiency traits measured on the same fish with these two methods, which although tedious, have the potential to be used for selective breeding of more efficient fish. As an example, using another feed efficiency trait, the residual feed intake (RFI), Silverstein (2006) found a significant correlation at family level between RFI of rainbow trout (*Oncorhynchus mykiss*) reared individually and RFI of fish reared in groups. He also detected differences among families for FI, growth, and RFI when fish were fed *ad-libitum* but not when fed a restricted ration. Besson et al. (2019) found a moderate but non-significant correlation between individual growth of European sea bass under restricted ration measured in isolated fish with growth of the same fish reared in groups under satiation. However, they found a relationship between individual FCR in fish reared in isolation with a restricted ration and subsequent *ad libitum* FCR in groups formed of the same fish. Given these variable results, it is important to determine whether both methods lead to similar FCR estimations or not, in order to help choose the most relevant methodology to set up selective breeding programs to improve feed efficiency.

The aim of the present study was to perform a comparison of data for traits relating to feed efficiency collected from Nile tilapia fed under different regimes, and to assess whether or not correlations were significant using different approaches. Growth, FI and FCR of individual Nile tilapia were compared when the same fish were held in small groups and fed either an optimal or restricted ration (half of the optimal ration), with FI being monitored using video-recording. Data collected from group-reared fish were also compared with those from the same fish reared in isolation on the same restricted ration, thereby testing the effects of group- and individual-rearing. Fish were reared over three consecutive periods of 7 days each under different feeding, recording and rearing scenarios: (i) in groups fed an optimal ration (g100) or (ii) fed a 50% restricted ration (g50), both with video recordings of all meals, and (iii) reared in isolation and fed a 50% restrictive ration. These comparisons were carried out in Malaysia on the Genetically Improved Farmed Tilapia (GIFT) strain, selected for 18 generations on growth by WorldFish (Ponzoni et al., 2011). The data from groups fed an optimal ration and from fish reared in isolation on a restricted ration were compared also in France in a second tilapia strain named “Cirad strain.” This additional test of the Cirad strain, which to our knowledge has not been selected for growth, provided a replication study to better assess the generality of the observations with the combination of a different strain (GIFT vs. Cirad), a different feed (Cargill vs. le Gouessant) and a different experimental countries (Malaysia vs. France).

## Materials and Methods

### Ethics Statement

This study utilized phenotypic data collected as part of the GIFT selective breeding program managed by WorldFish at Jitra, Kedah State, Malaysia (6°15′32°N; 100°25′47°E). All fish in the GIFT breeding population are managed in accordance with the Guiding Principles of the Animal Care, Welfare and Ethics Policy of the WorldFish including the “3-Rs” rule. Regarding the Cirad strain, this part of the study was carried out in accordance with the recommendations of Directive 2010-63-EU on the protection of animals used for scientific purposes. The protocols were approved by C2EA−36 (“Comité d'Éthique en Expérimentation Animale Languedoc-Roussillon”) under authorization APAFiS n° 2017112215278675 #12552 v4.

### Origin and Rearing of the Fish

The study was carried out on two distinct populations (GIFT and Cirad) in two different countries (Malaysia and France). The GIFT strain of Nile tilapia was selected for growth using a fully pedigreed design for 18 generations (Ponzoni et al., 2011). The families were produced by natural spawning from the 4th March to the 4th of April 2019 at the WorldFish Research station in Jitra, Kedah State, Malaysia (6°15′32°N; 100°25′47°E). The experiment was performed on 200 individuals from five families (40 fish per family) from the 10th of June to the 22nd of July 2019. After hatching, each family was reared in different hapas in the same pond and transferred to 1,500 L holding tanks (3 ×1 × 0.5 m) at 110 days post-hatching (dph). All the fish were injected with a Passive Integrated Transponder tag (PIT-tag, Trovan®) between 53 and 84 dph (around 10g of BW). Fish from each family were sorted according to their body weight to make four homogeneous groups of ten fish which were randomly put into four plastic aquaria of 60 L (61 × 30 × 33 cm). In total, 20 aquariums with ten fish in each were used. After anesthesia with clove oil (0.5 mL per liter of water), each fish was tagged in the dorsal muscle with two colored T-bar tags (Avery Dennison tags, 25 mm), one tag on each side of the body, using an Avery Dennison Mark III pistol Grip tool. This allowed each fish to be uniquely and individually identified by one color of tag within an aquarium regardless of which side of the body was shown and video recorded. Commercial pelleted feed (Cargill®, “Starter tilapia 6113”) with 34% of crude proteins, 5% of crude fat, 5% of crude fiber, and 12% of moisture was used to feed the fish during the whole experiment. Daily water temperature ranged from 28 to 30°C depending on the hour of measurement.

The Cirad strain of Nile tilapia was derived from a cross between Cirad-IRD females, originally from Egypt, kept in Cirad-IRD facility (Montpellier, France) for several generations and from males sold by FishGen (UK) in 2018 and kept in Cirad facilities in Palavas-les-Flots (France). This new cross was called “Cirad strain” to simplify the nomenclature for the present study. For this experiment, 320 fish from 16 families (20 fish per family) hatched from the 5th to the 26th of July 2019 were used. After hatching, each family was kept isolated until the end of the experiment. When fish reached on average 10g of BW, fish from each family were spread into two 38 L aquaria (10 fish per aquarium). After anesthesia with clove oil, each fish was tagged into the dorsal muscle with two colored T-bar tags (Avery Dennison tags, 25 mm), one tag on each side of the body, using an Avery Dennison Mark III pistol Grip tool. Each fish within an aquarium was tagged with an exclusive color to identify each fish individually regardless of which side of the body was shown and video recorded. Fish were fed a commercial pelleted feed (Le Gouessant, “Tilapia Starter Flot 1,” and “Tilapia Starter Flot 2”) with 38% of crude proteins, 8% of crude fat, 3.9% of crude fiber, and 7% of moisture during the whole experiment. Water temperature was maintained at 28°C during the whole experiment.

### Experimental Design and Trait Measurements

**Figure 1:**
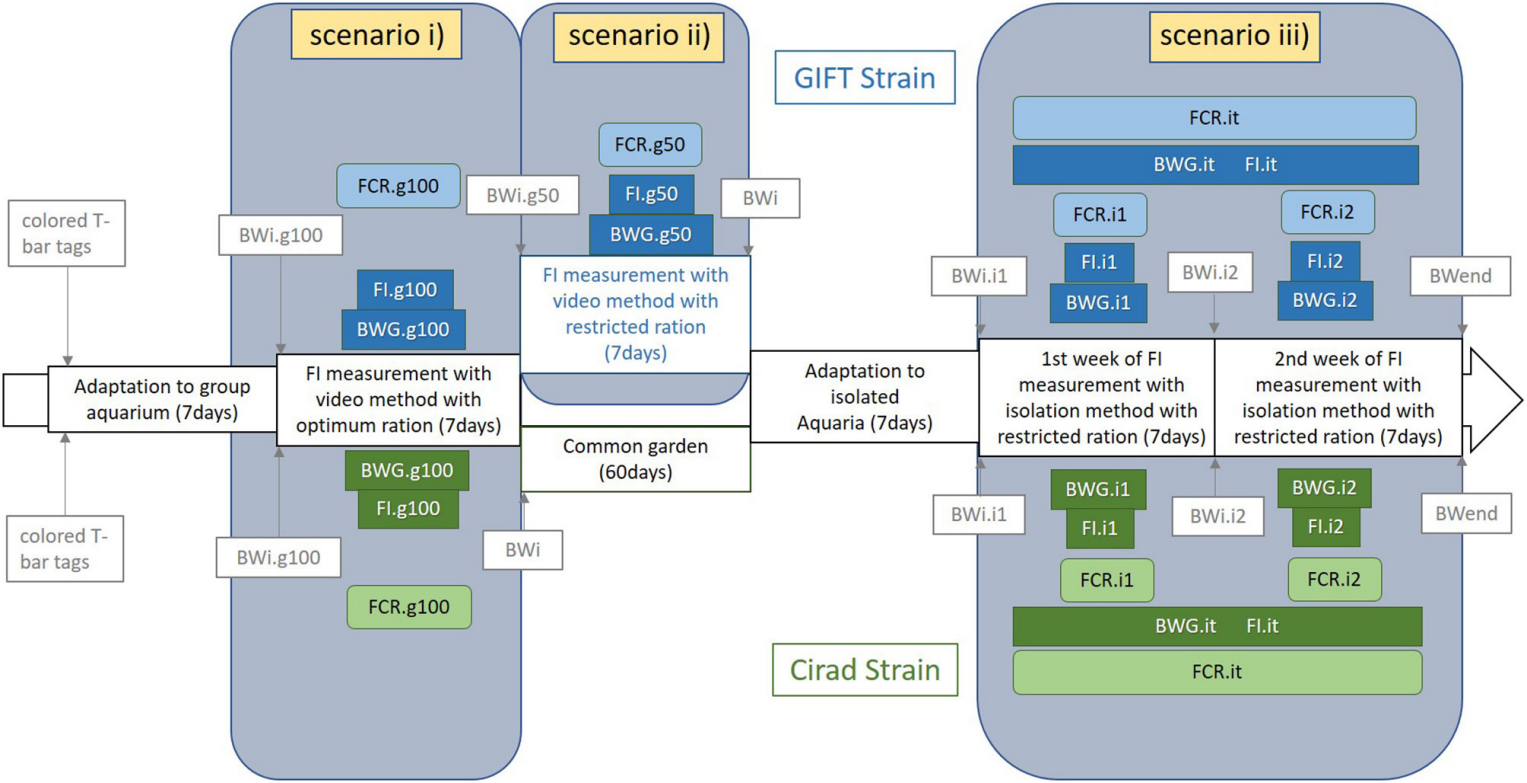
Scheme of the different scenarios designed in the experimental protocol and corresponding traits measured in each period for the GIFT strain (on the top of the figure and in blue) and the Cirad strain (on the bottom of the figure and in green). The three different scenarios were: (i) in groups fed an optimal ration (g100) or (ii) fed a 50% restricted ration (g50) both with video records of all meals and (iii) reared in isolation and fed a 50% restrictive ration. BWi, individual body weight; BWend, individual body weight at the end of the experiment; BWG, body weight gain; FI, feed intake; FCR, feed conversion ratio; .g100, fish reared in groups with 100% DFR ration; .g50, fish reared in groups with 50% DFR ration; .i1, .i2, and .it, fish reared in isolated aquaria during the first, the second, and the total of the first and second weeks of isolation period. The main measured traits were highlighted and the background of the frame was colored.

The experimental design is summarized in Figure 1. The experiment consisted of three periods of FI measurement, and consequently, three FCR measurement periods: (i) individual FI measured in groups (ten fish per group) with an optimal feed ration (coded g100), (ii) individual FI measured in groups (10 fish per group) on the same fish as (i) with half of the optimal feed ration (coded g50), (iii) individual FI measured in isolation on the same fish as (i) and (ii) with half of the optimal feed ration (coded i1, i2, and it for the first week of this period, the second week of this period and both weeks of this period together, respectively). In periods (i) and (ii), all the meals were video recorded. Fish were not measured in isolation with the optimal ration as they may waste too many pellets to allow precise counting, and the accuracy of the exact FI would thus be questionable. All fish were anesthetized with clove oil (0.5 mL per liter of water) when weighed during the course of the experiment. No sign of stress or abnormal behavior was seen during the experiment except the stress due to the normal fish interactions.

After 7 days of adaptation to group aquaria, all the individual fish were anesthetized and weighed (BWi.g100). In the first period of FI measurement fish were reared as previously described by de Verdal et al. (2017). To summarize, fish were fed twice a day with a 100% daily feed ration (DFR, in percentage of body weight) except the weighing day when they were not fed. The DFR was calculated based on the formula published by Mélard et al. (1997): *DFR* = 14.23 × *BW*^−0.322^ with BW the body weight of each fish (in g) at the beginning of each period (BWi.g100, BWi.g50, BWi.i1, and BWi.i2 were used to calculate the DFR used during the g100, g50, i1, and i2 periods, respectively, Figure 1). As different experimenters were involved in the feeding process, a calculated ration was preferred to an “*ad-libitum*” ration, which is less repeatable from one experimenter to another. This calculated ration was also useful to ensure that the same maximal feed ration was given at every meal. The DFR was equally shared for each of the two daily meals. Feed was given using two pipes going to the aquarium, allowing a reduction of stress since the fish did not see the experimenter when given the feed. Frequently, fish did not eat the entire DFR and the choice was made to stop the meal when a few pellets remained uneaten after ~1 min (corresponding actually to an *ad-libitum* ration). Uneaten pellets were removed from the aquarium using a small net.

All the meals were recorded by video for FI.g100 and FCR.g100 estimations. At the end of this first period of 7 days (12 meals), fish were anesthetised and weighed (BWi.g50) and the individual growth during that period (BWG.g100 = BWi.g50 − BWi.g100) was calculated.

In the second period of FI measurement of 7 days (12 meals) in groups, fish were fed a restricted ration (calculated as 50% of the DFR using the previously mentioned equation) to estimate the impact of a restricted ration compared to an optimal ration on FCR. As during the 100% DFR period, all the meals were video-recorded to count the number of pellets eaten by each individual fish and estimate FI.g50. At the end of this second period of 7 days (12 meals), fish were anesthetized and weighed, allowing calculation of BWG.g50 and estimation of FCR.g50.

Before the beginning of the third period, the 200 fish were randomly distributed into 200 10 L isolated aquariums and adapted for 7 days to this new individual rearing system. Each fish was able to see the fish in neighboring tanks. The third period consisted of 2 consecutive weeks with the same experimental protocol. All the fish were anesthetized and weighed at the beginning and the end of each week (BWi.i1, BWi.i2, and BWend), allowing calculation of BWG.i1 and BWG.i2. Fish were fed twice a day (except on the day of weighing) with 50% DFR, as in the second period. The DFR was updated every week for each fish. Feed for each individual fish was weighed accurately every day and the uneaten pellets were counted and removed from the aquaria at least 2 h after the last meal of the day. The uneaten feed weight was estimated assuming that all pellets had the same weight (16.2 ± 1.8 mg), and FI.i1 and FI.i2 were calculated for each week. Knowing the BWG and FI for both periods, it was possible to estimate FCR.i1 and FCR.i2 for the first and the second weeks of this third period of the experiment. To reduce the effects of FI fluctuations from 1 week to another, both weeks were combined and global estimations were done for BWG.it, FI.it, and FCR.it.

The same measurements were performed on the Cirad strain, except the measurement of FI in groups with restricted ration which was not performed due to logistical reasons (i.e., limited infrastructure availability), with the experiment undertaken from the 8th of October 2019 to the 16th of December 2019. The experiment was performed as described for the GIFT strain except that fish were fed 13 meals during the group period (an extra-meal was given the afternoon after weighing the fish). From the 320 fish measured in groups, a total of 133 randomly drawn fish were kept and measured for FI in isolation and were included in the analyses. Due to the limited number of aquariums available, fish were measured in three distinct batches (around 50 fish per batch). The experimental protocol for each batch was similar and the batch effect was not significant whatever the considered trait and consequently, was not included in the present analyses. In the meantime, fish were identified with a passive integrated transponder tag (PIT-tag, Biolog-id®) and reared in a common garden environment in four 300 L tanks for 5–6 weeks.

In both experiments, the number of fish was not sufficient to perform a genetic study, so the aim was to assess the phenotypic correlations between the measurement methods. The weekly FI was the sum of all the daily FI of the week. Mortality was recorded daily and the feed ration changed accordingly during the group rearing periods and dead fish data were removed for all analyses. Body weight gain (BWG) was calculated as the difference between the body weight of each fish at the end and at the beginning of the week. The feed conversion ratio (FCR) was calculated as the ratio between FI and BWG (FCR = FI/BWG), the most efficient fish being the fish showing the smallest FCR values.

The Kinovea 0.8.15 software (Copyright © 2006–2011—Joan Charmant & Contrib.) was used to analyse the videos of the meals and to count for the number of pellets eaten by each fish when reared in groups.

### Statistical Analyses

All statistical analyses were performed using the R software (R Development Core Team, 2018). Negative FCR (35 out of the 1,187 FCR measurements in total) values were not included in the statistical analysis. Outliers were highlighted using the boxplot.stats function of the R package “stats” (R Development Core Team, 2018) and were not included in the analyses. After checking with the Shapiro-Wilk test, data for several traits (mainly FCR) were not normally distributed even after several transformations and consequently, non-parametric tests were preferred for the data analyses. Wilcoxon tests were used to analyse the block effects (including the strain, experimental protocol, and feed used) when the same traits were measured in both conditions, to assess the consistency of the results. Spearman correlations between traits were estimated using the R package “psych” (Revelle, 2015).

## Results

### Basic Statistics

The Nile tilapia used in this study were at the juvenile stage (Table 1), with initial BW (BWi.g100) on average of 10.3 ± 2.6 and 11.2 ± 3.3 g for the GIFT and Cirad strain, respectively. The Cirad fish at the beginning of the isolation period were heavier than the GIFT fish (on average a difference of 4.2 g between both strains). The coefficient of variation of body weight was slightly higher for the Cirad strain (ranged from 27.5 to 41.0%) than for the GIFT strain (ranged from 23.0 to 26.9%, Table 1). The number of individuals in each family (from 1 to 28) and the number of families (five and 16 for the GIFT and the Cirad strain, respectively) were too small to consider this family level as relevant for the present analyses.

**Table 1:** Basic statistics: mean ± standard deviation, minimum, maximum, and coefficient of variation (CV) of all the traits measured during the experiment for the GIFT and the Cirad strain, and the *p*-value of the block effect calculated using Wilcoxon tests.

	**GIFT strain**				**CIRAD strain**				**Block effect**
	**Mean ± SD**	**Min**	**Max**	**CV**	**Mean ± SD**	**Min**	**Max**	**CV**	
BWi.g100	10.3 ± 2.60	5.20	16.5	24.7	11.2 ± 3.31	6.04	18.70	27.5	0.040
BWi.g50	14.6 ± 4.18	6.30	23.7	26.9	.	.	.	.	.
BWi.i1	21.1 ± 5.84	10.7	33.3	24.6	25.3 ± 10.6	9.98	54.9	4.00	0.007
BWi.i2	23.7 ± 6.01	12.5	36.3	23.0	29.1 ± 11.3	12.6	60.8	38.1	0.0004
BWG.g100	4.29 ± 1.64	1.10	8.30	37.0	3.03 ± 1.28	0.80	6.26	41.9	<0.0001
BWG.g50	1.60 ± 0.87	0.30	4.00	52.3	.	.	.	.	.
BWG.i1	2.97 ± 0.68	1.55	4.67	22.0	3.80 ± 0.99	1.69	6.47	25.7	<0.0001
BWG.i2	2.58 ± 0.54	1.30	3.95	19.6	4.22 ± 1.25	1.72	7.50	29.5	<0.0001
BWG.it	5.55 ± 1.07	2.85	7.91	18.4	8.02 ± 1.98	3.84	12.5	24.6	<0.0001
FI.g100	3.48 ± 0.97	1.50	5.87	26.5	2.27 ± 0.76	1.04	3.90	32.6	<0.0001
FI.g50	2.04 ± 0.61	0.91	3.64	28.2	.	.	.	.	.
FI.i1	3.02 ± 0.62	1.80	4.16	18.0	3.75 ± 1.07	2.03	6.45	28.0	<0.0001
FI.i2	3.35 ± 0.62	2.12	4.60	16.8	4.13 ± 1.10	2.37	6.92	25.9	<0.0001
FI.it	6.37 ± 1.22	3.92	8.76	17.4	7.87 ± 2.15	4.40	13.4	26.9	<0.0001
FCR.g100	0.87 ± 0.26	0.39	1.88	28.7	0.81 ± 0.22	0.40	1.44	27.5	0.041
FCR.g50	1.60 ± 0.86	0.48	4.00	52.7	.	.	.	.	.
FCR.i1	1.04 ± 0.20	0.68	1.59	18.9	1.00 ± 0.23	0.61	1.65	22.1	0.050
FCR.i2	1.33 ± 0.26	0.90	2.15	19.8	1.01 ± 0.18	0.67	1.48	17.8	<0.0001
FCR.it	1.16 ± 0.19	0.81	1.75	15.9	0.99 ± 0.14	0.73	1.32	13.9	<0.0001

*BWi, individual body weight; BWG, body weight gain; FI, feed intake; FCR, feed conversion ratio; .g100, fish reared in groups with 100% DFR ration; .g50, fish reared in groups with 50% DFR ration; .i1, .i2, and .it, fish reared in isolated aquaria during the first, the second, and the total of the first and second week of isolation period; ne, non-estimable*.

#### The GIFT Strain

During the restricted feeding period in groups, the BWG of GIFT fish was reduced and was to 37.3% of that of the same fish fed an optimal ration (Table 1). Feed intake during this restricted period was only reduced to 58.6% of the value observed with 100%DFR (from 3.48 to 2.04 g). Thus, FCR was lower in fish fed 100% DFR than in fish fed with 50% DFR. Interestingly, the coefficient of variation of BWG and FCR was higher when fish were fed under restriction than with an optimal ration (Table 1). Isolated GIFT fish showed similar growth, BWG, FI, and FCR during the first and the second week of measurement (Table 1). The coefficient of variation of BWG, FI and FCR was lower when fish were reared in isolation (ranged from 15.9 to 22.0%) than when they were reared in groups (from 26.5 to 52.7%).

#### The Cirad Strain

Because of the limited time infrastructure was available with the Cirad strain, it was only possible to compare FCR measured in groups with 100% DFR and in isolation. Therefore, we could not assess the specific effects of social interactions and feed ration on FCR but the comparison of the main results can be used to assess the replicability of some results with another strain and a different rearing protocol. Cirad strain fish reared in groups (on the optimal ration) had a lower FCR than in isolation (Table 1). It is interesting to note that whatever the trait, similar to the GIFT fish, coefficients of variation were higher when fish were reared in groups than they were in isolation.

#### Block Effect

The block effect, estimated using Wilcoxon tests (including the strain, site, experimental protocol and feed used) was always significant. Fish from Cirad strain were 8.7% bigger at the beginning of the group rearing period, and 19.9% heavier at the beginning of the isolated period than those of the GIFT strain (Table 1). The coefficients of variation of BWG and FI were higher for the Cirad strain than for the GIFT strain (Table 1).

### Phenotypic Correlations

**Figure 2:**
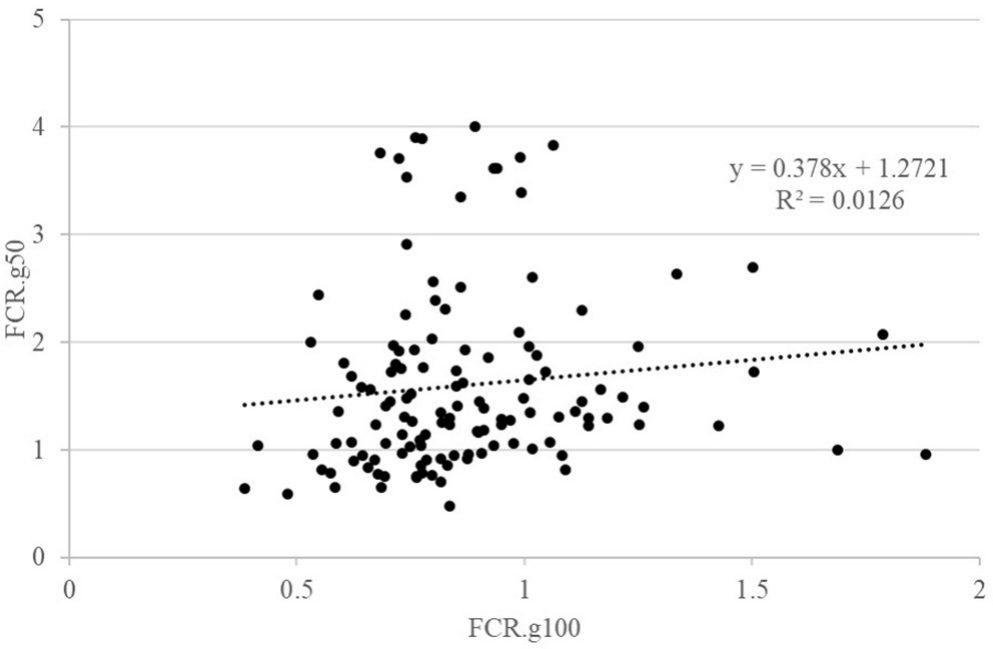
Relations between FCR when fish were reared in groups and fed with either an optimal feed ration (g100) or a 50% restricted ration (g50). Each point is representing data for one fish.

The details of the phenotypic correlations between the traits measured at all periods are presented in Table 2. The first question raised in this study was the impact of feed restriction on FCR in groups, which could only be estimated on the GIFT strain, as only those fish were subjected to a restricted feeding period in group rearing (Figure 2). The correlation between FCR.g100 and FCR.g50 was low and not significant (0.22) as illustrated in Figure 2. However, correlations were positive and significant, although not very high, between BWG.g100 and BWG.g50 (0.49) and between FI.g100 and FI.g50 (0.63).

**Table 2:** Phenotypic correlation between all the measured traits.

		**BWG**					**FI**					**FCR**				
		**g100**	**g50**	**i1**	**i2**	**it**	**g100**	**g50**	**i1**	**i2**	**it**	**g100**	**g50**	**i1**	**i2**	**it**
BWG	g100		**0.49**	**0.56**	**0.34**	**0.54**	**0.73**	**0.54**	**0.85**	**0.85**	**0.85**	**−0.65**	−0.25	0.19	**0.42**	**0.35**
	g50	.		0.30	0.18	0.29	**0.40**	**0.59**	**0.57**	**0.56**	**0.56**	−0.26	**−0.85**	0.17	0.28	0.25
	i1	**0.40**	.		**0.54**	**0.91**	**0.49**	0.24	**0.61**	**0.69**	**0.65**	−0.29	−0.23	**−0.55**	0.06	**−0.32**
	i2	0.25	.	**0.56**		**0.83**	0.27	0.08	**0.39**	**0.43**	**0.41**	−0.25	−0.15	−0.24	**−0.60**	**−0.48**
	it	**0.36**	.	**0.85**	**0.90**		**0.44**	0.21	**0.59**	**0.66**	**0.63**	**−0.33**	−0.23	**−0.47**	−0.24	**−0.44**
FI	g100	**0.79**	.	**0.42**	0.25	**0.35**		**0.63**	**0.70**	**0.71**	**0.71**	0.00	−0.10	0.11	**0.36**	0.26
	g50	.	.	.	.	.	.		**0.50**	**0.49**	**0.50**	−0.11	−0.11	0.16	**0.33**	0.28
	i1	**0.51**	.	**0.70**	**0.81**	**0.86**	**0.49**	.		**0.99**	**1.00**	**−0.46**	**−0.37**	0.27	**0.44**	**0.41**
	i2	**0.52**	.	**0.74**	**0.81**	**0.87**	**0.50**	.	**1.00**		**1.00**	**−0.46**	**−0.37**	0.18	**0.42**	**0.33**
	it	**0.52**	.	**0.72**	**0.81**	**0.86**	**0.50**	.	**1.00**	**1.00**		**−0.46**	**−0.37**	0.22	**0.43**	**0.37**
FCR	g100	**−0.66**	.	−0.09	−0.08	−0.11	−0.10	.	−0.22	−0.22	−0.22		0.22	−0.17	−0.20	−0.22
	g50	.	.	.	.	.	.	.	.	.	.	.		−0.08	−0.13	−0.12
	i1	0.20	.	−0.22	**0.39**	0.14	0.16	.	**0.50**	**0.45**	**0.47**	−0.18	.		**0.41**	**0.86**
	i2	0.31	.	0.12	**−0.49**	−0.25	0.30	.	0.07	0.08	0.08	−0.15	.	0.00		**0.80**
	it	**0.35**	.	−0.11	−0.05	−0.08	0.29	.	**0.41**	**0.37**	**0.39**	−0.24	.	**0.73**	**0.65**	

*GIFT strain correlations are above the diagonal, and Cirad strain correlations are below the diagonal. Bold values are significantly different from zero*.

*BWG, body weight gain; FI, feed intake; FCR, feed conversion ratio; .g100, fish reared in groups with 100% DFR ration; .g50, fish reared in groups with 50% DFR ration; .i1, .i2, and .it, fish reared in isolated aquaria during the first, the second, and the total of the first and second week of isolation period*.

**Figure 3:**
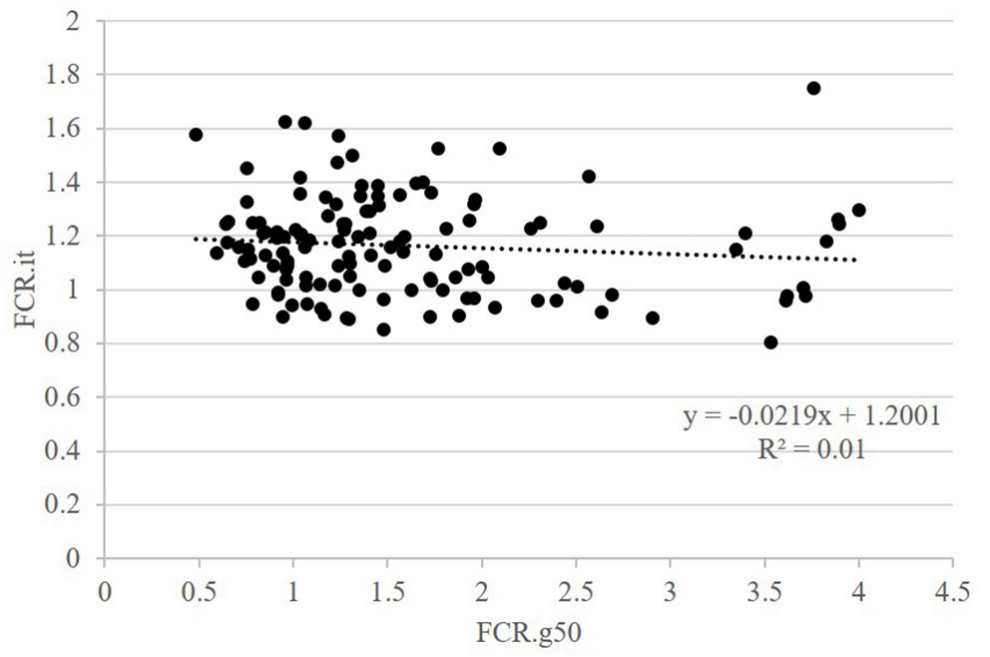
Relations between FCR when fish were fed a restricted feed and reared in groups (g50) or in isolation (it). Data for GIFT only. Each point is representing data for one fish.

**Figure 4:**
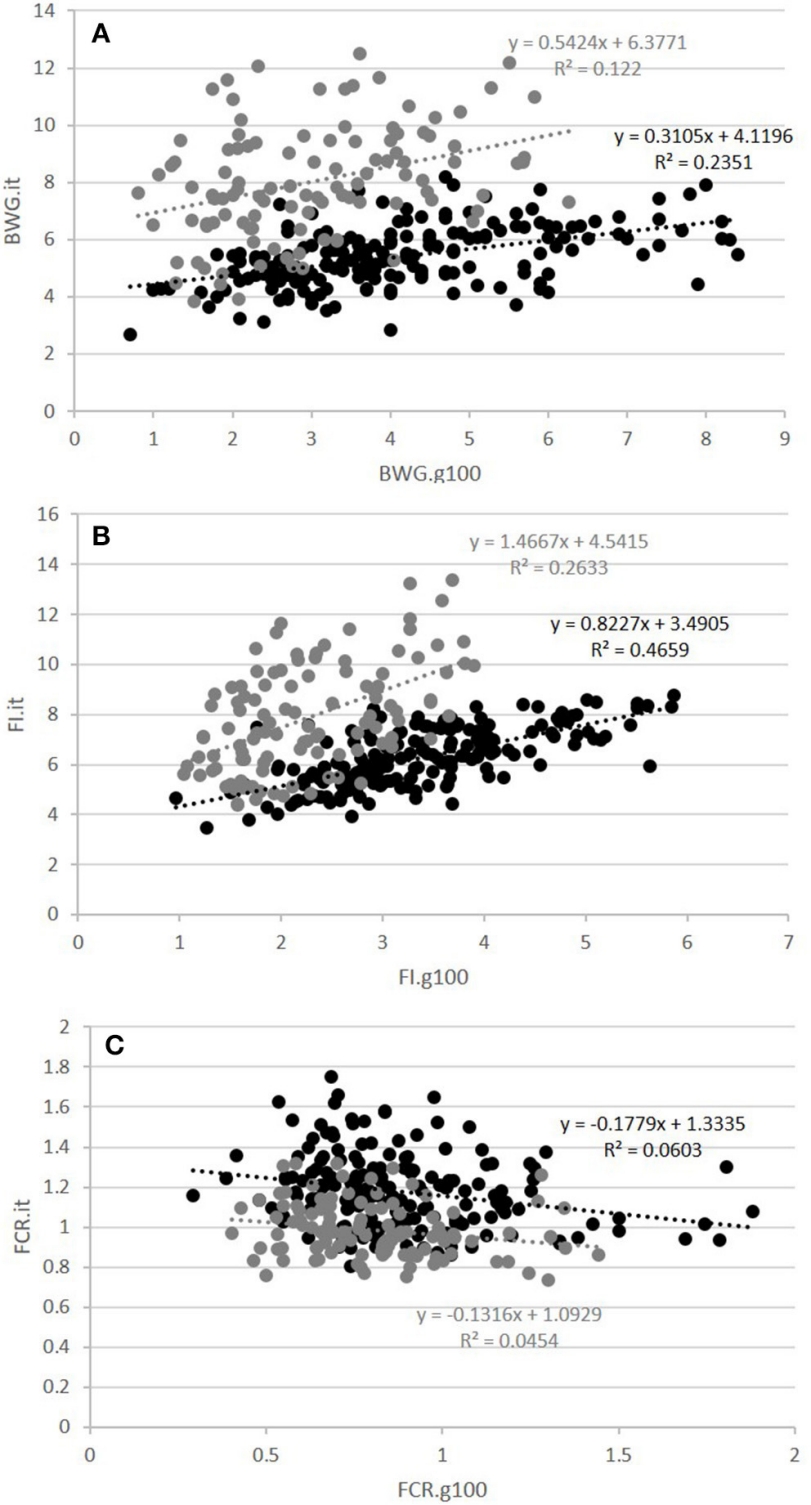
Relations between fish reared in groups and fed with an optimal feed ration (g100) and fish reared in isolation with a restricted ration (it) for BWG **(A)**, FI **(B)**, and FCR **(C)**. Black and gray circles corresponded to individuals of the GIFT and Cirad strains, respectively. The equation of the linear regression and the coefficient of determination R^2^ are surrounded in black and gray for the GIFT and the Cirad strain, respectively.

The second question raised was whether FCR measured in groups was correlated with FCR measured on isolated fish. This was done on the GIFT strain only with restricted ration (Table 2 and Figure 3). The correlations between FCRs measured in groups with restricted feeding (FCR.g50) and FCRs measured in isolation (FCR.i1, FCR.i2, and FCR.it) were low, negative (from −0.13 to −0.08) and not significant (Table 2). Here again, positive and significant correlations were seen between FI.g50 and FI.it (0.50) but this time not between BWG.g50 and BWG.it (0.29, NS). Comparison between fish reared in groups fed with an optimal ration (video method) and fish reared in isolation and fed with a 50% restricted ration (isolation method) was possible both for the Cirad and for the GIFT strain (Figure 4). In both strains, BWG.g100 and BWG.it were significantly correlated (0.54 in the GIFT strain, 0.36 in the Cirad strain), as well as FI.g100 and FI.it (0.71 in the GIFT strain, 0.50 in the Cirad strain). Additionally, BWG was significantly correlated to FI in both periods, with higher correlations for the GIFT strain (0.85) than for the Cirad strain (0.52, Table 2). Here again, FCRs measured in groups with optimal ration and in isolation with restricted ration were not significantly correlated (correlations of −0.17 and −0.18 for GIFT and Cirad strain, respectively).

## Discussion

In selective breeding programs, it is essential to measure accurately the trait under selection. No method is available to accurately measure the individual FI of fish reared in large groups (in tanks or ponds) during several consecutive days. The only two methods employed to date for genetic studies to precisely measure the individual FI of many fish during several consecutive days are group rearing with video-recording of all the meals and *a posteriori* analysis of all the videos (de Verdal et al., 2017) or individual rearing with a restricted ration (Besson et al., 2019). The objectives of the present study were to assess the impact of ration level (100% DFR or 50% DFR) and of the rearing system (group rearing or isolation) on FCR, trait commonly used to assess feed efficiency.

### Impact of Feed Restriction

Growth and FI were significantly and positively correlated when measured in groups with an optimal or a restricted ration. However, FCRs measured in both conditions were poorly to moderately correlated (0.22), and the correlation was not significantly different from zero. Consequently, in groups, the most efficient fish fed with an optimal ration were not always the most efficient fish when fed with a restricted ration. Using group measurements in rainbow trout, Azevedo et al. (1998) and Rasmussen and Ostenfeld (2000) found a restricted feed ration had a significant effect on growth (fish under the restricted ration growing less than under high ration) but did not impact feed efficiency. In the present experiment, BWG and FCR in group reared fish were more variable when fish were fed with 50% DFR (CV = 52.3 and 52.7%, respectively) than when fish were fed with 100% DFR (CV = 37.0 and 28.7%, respectively) but the CV of FI did not change between these two periods. Using X-Ray methodology, Jobling and Koskela (1996) showed a similar increase in the CV of BWG under restricted feeding in rainbow trout, which in their case was also accompanied by an increase in the CV of FI. They attributed this to an increase of the social interaction when feed is restricted, which could also partly be the case here, although no increased variability in FI was seen. With fish fed an optimal ration, de Verdal et al. (2019) did not find any correlations between agonistic behavior and growth and feed efficiency. However, it can be hypothesized that agonistic behaviors were exacerbated under restricted diets, and consequently some fish will lose more energy to deal with these social interactions than others.

In rabbits and pigs selection for growth using a restricted feed ration was shown to improve feed efficiency of their progenies even when they were held in conditions where they were fed to satiety (Nguyen et al., 2005; Drouilhet et al., 2016). The proposed explanation of these results is that higher growth under a restricted diet is due to lower maintenance requirements, which is also beneficial for animals fed to satiety. The maintenance requirements of fish, as poikilotherms, cannot be easily compared to those of warm blooded livestock species, which may explain some differences observed between fish and livestock. In the present study, the phenotypic correlations were high (as in livestock species) between BWG.g50 and FCR.g50, but not between BWG.g50 and FCR.g100, indicating that selection for growth under restricted feeding in groups was unlikely to improve feed efficiency in fish fed to satiation. In rainbow trout, it was shown that feeding a restricted ration created social hierarchies in the tanks, leading to some fish consistently eating a larger or smaller share of the ration given, which was less the case under satiation (McCarthy et al., 1992; Jobling and Koskela, 1996). Then, it seems reasonable not to select fish for growth under restricted feeding in groups to improve feed efficiency. Nevertheless, our results were based only on phenotypic correlations, which can influence conclusions considerably. Firm conclusions on this issue will require the estimation of genetic correlations, but using a much larger number of fish.

### Impact of the Social Interactions

Individual rearing systems remove all the direct social interactions between fish whereas clear social interactions were seen in videos of group reared fish, including an extensive repertoire of agonistic behaviors between fish (de Verdal et al., 2019). There were no significant correlations between FCR of GIFT strain reared under restricted ration when measured in groups (g50) or in isolation (i1, i2, or it). Using exactly the same experimental setting up with the video analyses method, de Verdal et al. (2019) noted that neither the amount of agonistic behaviors nor the hierarchy measured outside the meals in Nile tilapia were significantly correlated with feed efficiency when fish were fed with 100% DFR. These results would suggest there should be limited or no effects of social interactions. However, the present experiment shows a clear effect of group rearing on the FCR estimations. The experiment of de Verdal et al. (2019) only measured agonistic behavior but social interactions are broader and the present results suggest more complex interactions are involved. A number of studies have reported that fish reared in isolation were more efficient (Jackson et al., 2003; Silverstein, 2006) as a result of stress reduction. It is known that stress, by increasing the maintenance requirements, reduces the efficiency of the fish to convert feed (Martins et al., 2006, 2011). From the present data, GIFT fish reared under restricted feeding in groups (g50) showed a FCR 37.9% higher than when reared in isolation (it). It is important to note that under our feed ration conditions, the coefficient of variation of FCR of fish reared in groups was almost twice that of the same fish reared in isolation. Group rearing could induce stress at the individual level, with a probable high variation between dominant and subordinate fish (Martins et al., 2005, 2006). This social impact, leading to an increased energy expenditure, could explain the differences in CVs of FCR between fish reared in groups or in isolation and why the most efficient fish were not the same when the rearing conditions changed. An interesting aspect of our study is also the fact that the correlation between FI in isolation under restricted feeding and FI in groups is higher when group results are obtained under satiation than when they are obtained under restricted feeding (*r* = 0.71 vs. 0.50). Similar observations are made with BWG (*r* = 0.54 vs. 0.29). This probably highlights, as discussed before, that social hierarchies are very high in groups under restricted feeding, and that social interactions are less intense both in individual rearing and in groups fed to satiation, in accordance with the results of de Verdal et al. (2019) estimating non-significant phenotypic correlations between FCR and agonistic behaviors in juvenile Nile tilapia reared in groups and fed to satiation. Still, although both BWG and FI are more correlated between group satiation and isolation than between groups under restricted feeding and isolation, this does not lead to significant correlations of FCR between both methods.

These results are probably dependent of the fish species under consideration. Nile tilapia is known to be a social species, with behavioral interaction between fish, which is not the case for all fish species. As a consequence, the difference of stress experienced by a Nile tilapia reared in groups or in isolation will not be comparable with other species, which may explain the different results found in the literature. Strand et al. (2007) indicated that juvenile perch (*Perca fluviatilis*) were much more efficient in large groups (FCR of around 1.1 when reared in groups of 12 fish) than in isolation (FCR of around 4.5) probably due to reduced stress when fish were reared in groups. Besson et al. (2019) also showed that FCR of European sea bass reared in individual aquaria was higher (1.38) than that of the same fish held in groups (~1.23). Taken as a whole all these results tend to show that the individual efficiency of fish reared in groups or in isolation differs, depending probably on the differences in stress levels experienced by the fish according to the rearing conditions and species.

### Choice of Method for Use in Selective Breeding Programs

The final aim of the present work was to assess which methodology might be best in a selective breeding program targeting feed efficiency (through FCR) as one of the breeding objectives. To succeed in a selective breeding program, it is essential to have an accurate measure of the phenotype of interest, and the trait should also ideally be measured in conditions similar to commercial production to reduce the risk of genotype by environment interactions. Nile tilapia is produced in large groups in ponds/cages/tanks/raceways where social interactions occur. As the measure of FCR in groups and in isolation are not significantly correlated in the present study, selecting fish in groups seems more relevant in the case of the tilapia than measuring fish in isolation. As discussed in the preceding section this is likely not true for all fish species. As an example, Besson et al. (2019) showed that the most efficient European sea bass measured in isolated aquaria tended to stay the most efficient later in life when reared in groups. One of the main advantages of the isolation method compared to the video method is the fact that the phenotypes are known immediately, whereas using the video method requires time-consuming video analysis in order to estimate the phenotypes. However, both methods involve a large amount of phenotyping work which may restrict the number of individuals and families that can realistically be evaluated.

The high CV of FCR when fish were fed under restriction could be seen as an interesting feature for a selective breeding program, as the level of phenotypic variance is one of the criteria to take into consideration when choosing the best trait for which to select, with higher variances being preferred (Falconer and MacKay, 1996). However, we discussed that selection under a restricted ration may increase agonistic behavior between fish, which would not be favorable in production systems, and could increase mortality in the farms. Those effects could be enough to outweigh the benefit of selecting from a higher observed variance. Furthermore, it was previously shown that agonistic behaviors were negatively correlated with growth when fish were reared in an environment where the level of social interactions was high (Ruzzante and Doyle, 1991). Thus, selecting fish for feed efficiency in groups under restricted feeding is likely not a valuable option.

There is no perfect method to measure FI accurately over several days and to estimate FCR robustly. However, rearing different tilapia strains in different conditions (experimental protocols, feed, and country) gave similar conclusions: there is no significant phenotypic correlations between the tested methods to measure accurately FI in Nile tilapia. This suggests some level of generality of the observations done. The aim of the present study was to compare two methods used to estimate accurately individual feed efficiency in Nile tilapia during several consecutive days and to highlight the most relevant method to use in selective breeding programs. To be sure the results undoubtedly meet our objective, it would be necessary to develop an experiment comparing these methods at the genetic level, including a much larger number of fish. Phenotypic correlations do not allow to predict how traits are correlated at the genetic level and what would be the impact of the measurement method in a selective breeding program.

The most favorable outcome would have been to see good correlations between FCR measurements done with the group or with the isolation method, which would have given more opportunities for designing breeding programs for feed efficiency. This was not the case, and then there is no simple answer to guide the choice of the method. What is relatively clear is that the group method under restricted feeding is not adequate, as it exacerbates social hierarchies, and it is not representative either of the *ad libitum* group method or of the isolation restricted method. As the question is complex, selection experiments will be needed to ascertain which are more efficient and economically viable phenotyping methods for selective breeding for feed efficiency. The global aim is to have more efficient fish in a conventional farming environment (i.e., ponds). It could therefore be suggested to phenotype fish in aquariums (by video) and to place them in ponds according to their individual feed efficiency and then to evaluate their FCR with a specific feed ration when raised in ponds. Having ponds with “efficient” fish and ponds with “non-efficient” fish would allow the impact of selection in aquariums to be evaluated. The same experiment could be carried out using FCR values measured with isolated and restricted fish to sort the fish.

## Data Availability Statement

The raw data supporting the conclusions of this article will be made available by the authors, without undue reservation.

## Ethics Statement

The animal study was reviewed and approved by C2EA−36 (Comité d'Éthique en Expérimentation Animale Languedoc-Roussillon) and Guiding Principles of the Animal Care, Welfare and Ethics Policy of the WorldFish including the 3-Rs rule.

## Author Contributions

CR and HV designed the experiment. CR, TT, VD, MC, and HV performed the experiment. CR, MV, and HV analyzed the data. CR, MV, TT, JB, and HV wrote the paper. All authors read and approved the final manuscript. All authors contributed to the article and approved the submitted version.

## Conflict of Interest

The authors declare that the research was conducted in the absence of any commercial or financial relationships that could be construed as a potential conflict of interest.
